# Thermal transport analysis of six circular microchannel heat sink using nanofluid

**DOI:** 10.1038/s41598-022-11121-y

**Published:** 2022-05-16

**Authors:** Hassan Waqas, Shan Ali Khan, Umar Farooq, Taseer Muhammad, Ahmad Alshehri, Sumeira Yasmin

**Affiliations:** 1 Department of Mathematics, Government College University Faisalabad, Layyah Campus, Layyah, 31200 Pakistan; 2 Department of Mathematics, College of Sciences, King Khalid University, Abha, 61413 Saudi Arabia; 3 Department of Mechanical Engineering, HITEC University Taxila, Taxila, 47080 Pakistan; 4 Department of Mathematics, Faculty of Science, King Abdulaziz University, Jeddah, 21589 Saudi Arabia

**Keywords:** Energy science and technology, Engineering, Materials science, Mathematics and computing, Nanoscience and technology, Optical techniques

## Abstract

Electronics devices growth in the last decade of the twentieth century ushered in a revolution inside the electronics segment. Continuous micro-sizes and operation cause these devices to heat up, resulting in a reduction in their performance or damage to their parts. Because heat can decrease device performance and life span while also wasting energy, offering an incorporated and effective cooling system has become a significant part of the design of device equipment. One of the key challenges of modern generation technology is the cooling of electronic devices. Nanofluids have attracted attention in a broad range of engineering implementations due to their great properties, which may be used to effectively cool devices while also improving energy efficiency. In view of the above defects, this numerical research object to examine the chip surface temperature, heat transfer rate, thermal resistance, Darcy friction factor and reliability of microelectronic chips in minichannel heat sinks is scrutinized by utilizing a ${\text{TiO}}_{2}$/water nanofluid as a coolant and comparing the nanoliquid outcomes with the outcomes of water. ${\text{TiO}}_{2}$/Water nanofluids at 1%, 2% and 3% volume concentrations are employed for this scrutinization. Here, a commercial CFD ANSYS (R19.2) FLUENT software package is used to analyze the electronic chip performance. The CFD ANSYS (R19.2) FLUENT software package is used for modeling, meshing and simulation of the current study.

## Introduction

Currently, modern electronic machinery generates strong heat fluxes. As a result, advanced electronic machinery, such as integrated circuits, electronic chips and computer microprocessors, demands large thermal dissipation rates for cooling. Heat sinks are technologies to remove heat generated from electronic processors with the help of the energy difference between the warming surface and the coolant. The objectives of using heat sinks are divided into two categories: (I) to improve the rate of heat dissipation to improve the efficiency of electronic devices and (II) to increase the electrical package dependability and performance. It is generally acknowledged that electronic chip malfunction and excessive energy consumption are caused by shrinking electronic component dimensions, which drastically boosts heat flux per unit area, resulting in heat difficulties and deterioration in chip efficiency and reliability. One of the most difficult problems in the creation of next-generation circuit boards, microprocessors, and other tiny integrated memory chips is addressing these challenges. Several kinds of heat exchangers and cooling processes are currently used in electrical cooling. Electronic cooling can be accomplished using a variety of approaches, including jet impingement cooling, pool boiling, two-phase flow in microchannels, spray cooling, and heat pipes. In the past, air-based cooling methods were widely used. However, due to the poor thermal transportation properties of air, they have some drawbacks^1,2^. Water-based strategies were later used to overcome the drawbacks of the air cooling process. According to many researchers, the thermal efficiency of water-based systems is significantly better than that of air-based systems. However, because of the latest developments in micro- and nanoscale heat transfer components, the heat efficiency of water-based systems may be limited in their ability to eliminate large heat fluxes from advanced electronic devices. Li^3^ proposed a nanofluid flow through microchannel. Paisarn and Lursukd^4^ examined the heat sink impact on minirectangular fins to improve the thermal performance through nanofluids. The output demonstrated that the thermal efficiency rate for nanoliquids is greater than that for deionized water. Farsad et al.^5^ scrutinized the numerical computations of microchannel heat sinks utilizing nanofluids and found that the microchannel heat sink cooling performance was better than that of pure water in minichannel heat sinks. Saadoon et al.^6^ explored the thermal transportation of nanofluids in minichannel heat sinks.

Although common working fluids have poor thermal characteristics, it appears that using fluids with improved heat characteristics rather than ordinary fluids is required^7,8^. Solids, on average, have stronger heat conductivities than base liquids^8^. Therefore, it appears that scattering solid particles in host fluid may improve the heat proficiency of host fluid^9,10^. Tiny-sized particles are unstable and sediment, whereas Choi^11^ demonstrated that tiny-sized particles disperse faster than micron-sized particles. These nanofluid suspensions have greater heat conductivities than the base liquid^12^. Muhammad et al.^13^ investigated the numerical computations of Eyring-Powell nanofluids over three-dimensional surfaces with Arrhenius activation energy. Sheikholeslami et al.^14^ examined the thermal performance impacts on copper/water nanoliquids by adopting CVFEM. Wakif et al.^15^ investigated magneto nanofluid flow through horizontal layers. Khan et al.^16^ discussed the behavior of Casson blood base nanofluid flow through a rotating disk.

In various areas, nanofluids with high heat efficiency have been commonly utilized rather than conventional media (such as water and oil) to fulfill the demand of high-performance heat transfer media. Currently, nanoliquids have been recommended by the majority of researchers in the field of solar energy. Yu et al.^17^ suggested that CuO/Ag nanoliquids with a volume of 0.025 percent have the best photothermal renovation capacity. This helps to improve the transfer of energy between light and heat in the solar heat collector packed with nanoliquids. Moghadam et al.^18^ discussed copper oxide/water nanofluid flow through plates inside a solar collector.

Computational fluid dynamics (CFD) approaches are strong tools for simulating fluid flow and associated heat and solutal transport by computing a numerically mathematical system that governs these processes, taking use of rapid and ongoing advances in computers and computing methodologies. Extensive comprehensive examination, fundamental investigations of redesign and new systems, in-depth product research & innovation, and troubleshooting are all areas where CFD simulations are useful^19^. In comparison to analytical and experimental fluid dynamics, CFD is highly significant in computations of microelectromechanical system (MEMS) technologies, particularly in the design of effective microchannel heat sinks. Adopting CFD modeling tools in invention and design saves time and cost when compared to experimental techniques. Klazly and Bognár^20^ analyzed the CFD computations of nanofluid flow influenced by flat plates. Mohammed et al.^21^ used a rectangular tube to analyze nanofluids. Naphon and Nakharintr^22^ examined the numerical computations of nanofluids in microchannel heat sinks.

The fast demand for electronic equipment necessitates the development of small advanced cooling technology that can deliver higher performance and reliability. Electronic equipment faces challenges such as excessive power consumption and short life. Many electronic cooling strategies are being introduced by researchers. One of them is a desire to learn more about electronics cooling. Many scientists have scrutinized the significance of several nanofluid combinations on the cooling performance of electrical devices. The advent of current technology has resulted in a reduction in the dimensions of industrial equipment in recent years. However, adequate cooling and energy removal is a major issue in utilizing such equipment, especially in electronic systems. As a result, improving the thermal efficiency of systems has become extremely important. Based on the abovementioned survey of the literature, a few analyses have focused on circular microchannel heat sinks with fins by utilizing water-based suspensions with ${\text{TiO}}_{2}$ nanoparticle applications for heat transfer in electronic chips. Therefore, to fill this gap of investigation, we tried to improve the cooling proficiency of electronic chips in regular six-channel heat sinks by using a ${\text{TiO}}_{2}$/water nanofluid utilizing the numerical finite volume method. The main purposes are mainly as follows:

- The impacts of the heat transfer rate, Nusselt number, Darcy friction factor, thermal resistance and reliability of the electronic chip on the microchannel heat flux are scrutinized.
- The effects of the surface temperature of the chip and ${\text{TiO}}_{2}$/water nanofluid behavior as a coolant are investigated.
- Here, the cooling efficiency of nanofluid $\phi = 1\% ,\phi = 2\% ,\phi = 3\%$ in a microchannel heat sink is scrutinized numerically by utilizing CFD ANSYS-Fluent.
- The modeling, meshing and simulation are explored by using computational CFD ANSYS-FLUENT (R19.2).
- The flow governing system of equations is approached numerically by adopting the finite volume method (FEM) in ANSYS-FLUENT (R19.2).

## Mesh specifications/generation

### Mesh independence study

Throughout the mesh independence study, three different cases were carried out: (I) coarse, (II) normal and (III) fine. After the analysis, we found a fine mesh to be given accurate results and of good quality. The mesh characteristics are reported in Table 1. Eight dissimilar grids were utilized to confirm that outcomes were not dependent on the grid. The Nusselt number and Heat transfer outcomes (depicts in Table 2) are utilized as the indicators of the outcomes. Due to diverse outcomes, the fifth grid was preferred as final grid. Reasons for choosing a fifth grid are summarized below:

- Better and smoother solution convergence at good criteria.
- Much better accuracy.
- Structured multi zone mixed cells mesh along with much better quality as compared to other meshes.
- Feasible Results in relatively less computational time.

**Table 1:** Mesh features.

Number of elements	Number of nodes	Maximum skewness	Minimum OQ	Mesh quality
421,945	174,216	0.75999	0.29128	Good

**Table 2:** Evaluation of Nusselt number and heat transfer coefficient for different grid resolution.

Mesh type	Nodes	Elements	Heat transfer coefficient	Nusselt number
Mesh 1	9778	22,808	3137.23	29.1835
Mesh 2	12,475	28,655	3137.58	29.1868
Mesh 3	40,216	94,904	3700.45	34.4228
Mesh 4	63,600	152,503	3920.88	36.4733
Mesh 5	174,216	421,945	4511.87	41.9709
Mesh 6	227,293	529,703	3780.31	35.1657
Mesh 7	291,736	700,021	3646.63	33.9221
Mesh 8	1,412,456	634,481	3647.35	33.9288

For current simulation purposes, a machine with the following specification is utilized:

1. Ram: 12 GB.
2. Processor: Intel core i5.
3. Time: 30 min iteration 100.
4. Convergence at 100 iteration.

**Figure 1:**
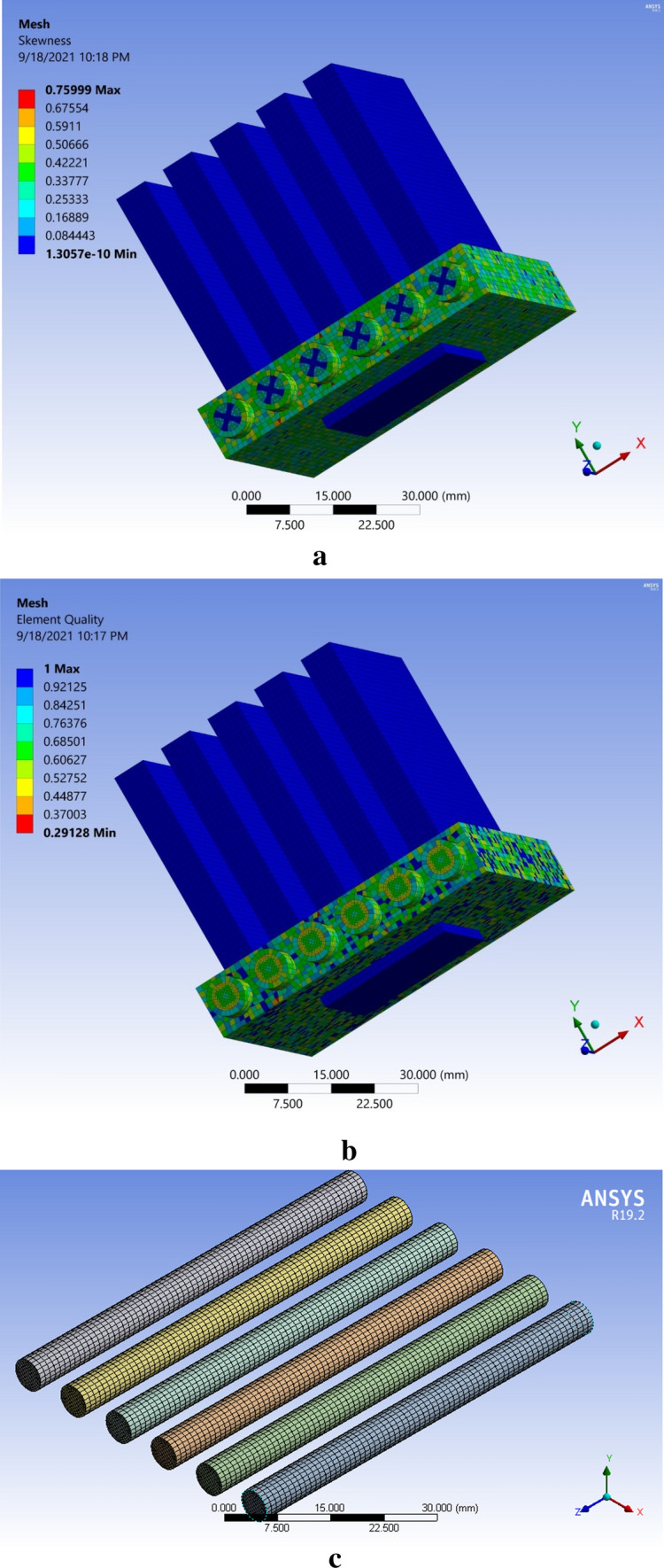
Mesh sketch, (**a**) skewness, (**b**) element quality, and (**c**) mesh of six microchannels.

Meshing of the microchannel with a heat sink was performed with the help of a commercial ANSYS-FLUENT (R19.2) tool. Figure 1 shows the mesh of the microchannel heat sink in cases of skewness, element quality and mash of six microchannels.

## Methodology

### Problem description

**Figure 2:**
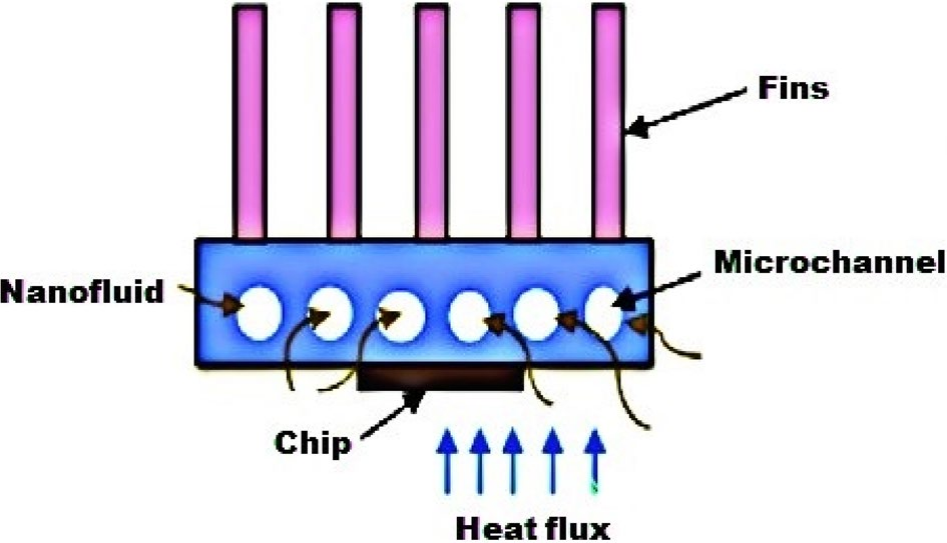
Minichannel heat sink schematic diagram.

**Figure 3:**
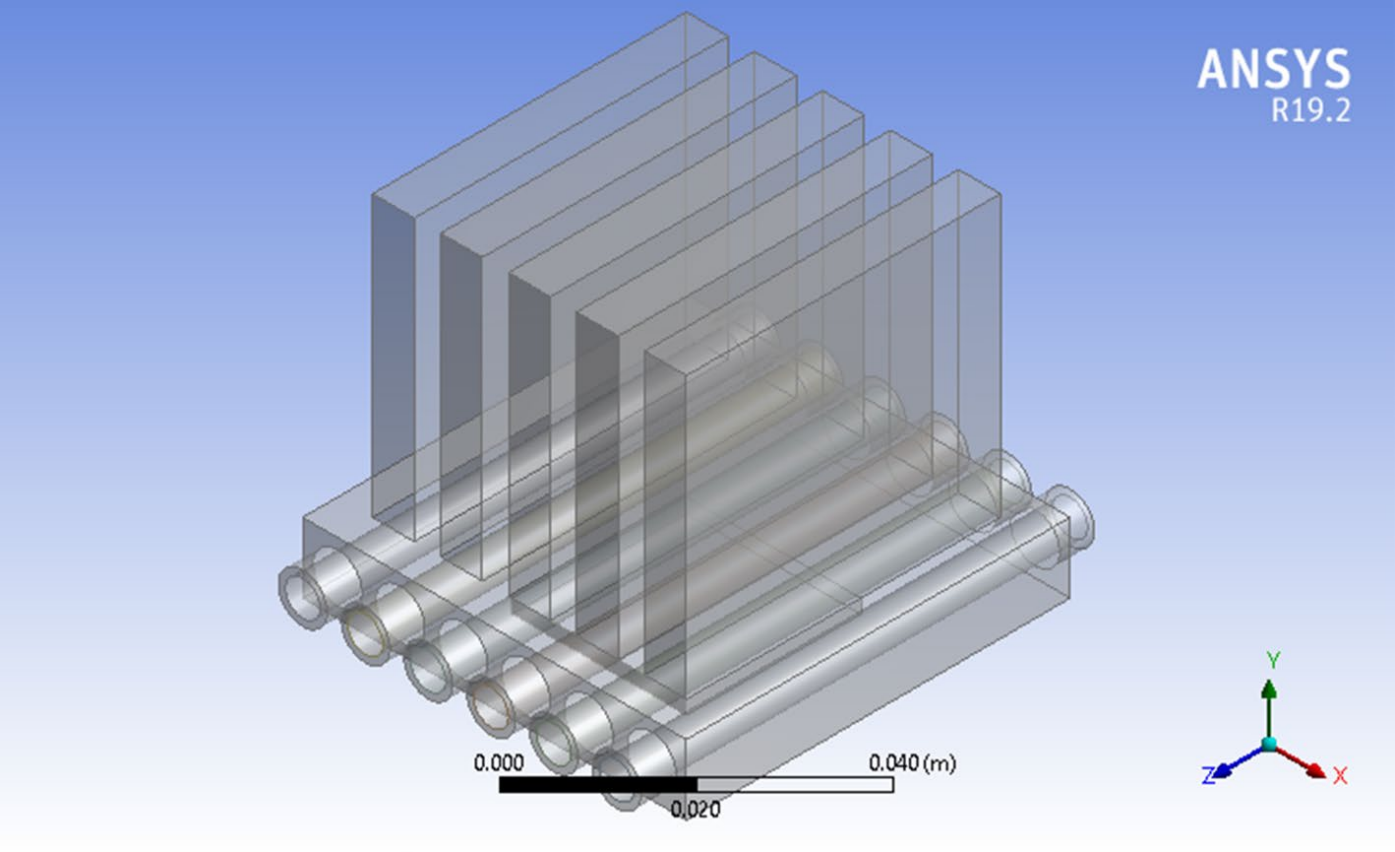
Microchannel heat sink geometry view created by ANSYS.

**Figure 4:**
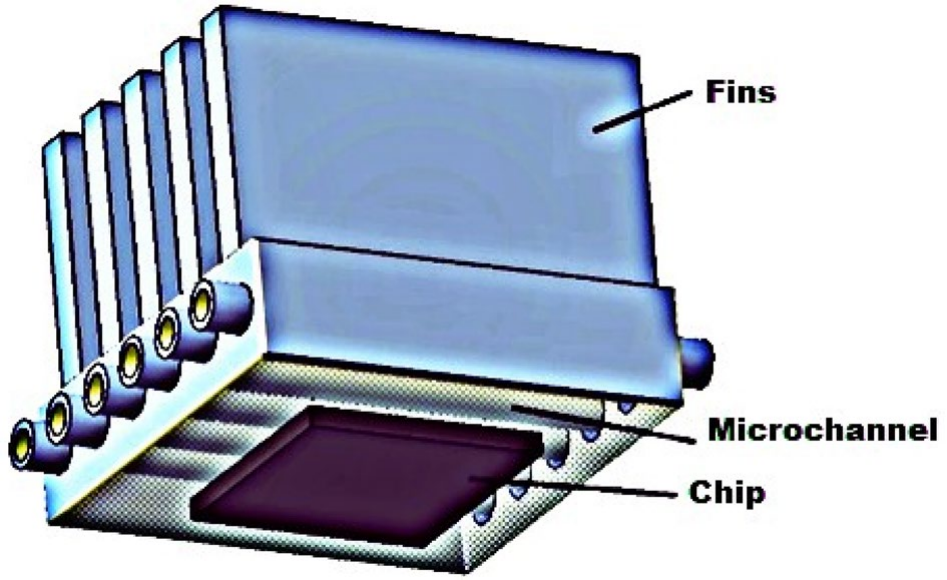
Geometry of Micro channel heat sink.

**Figure 5:**
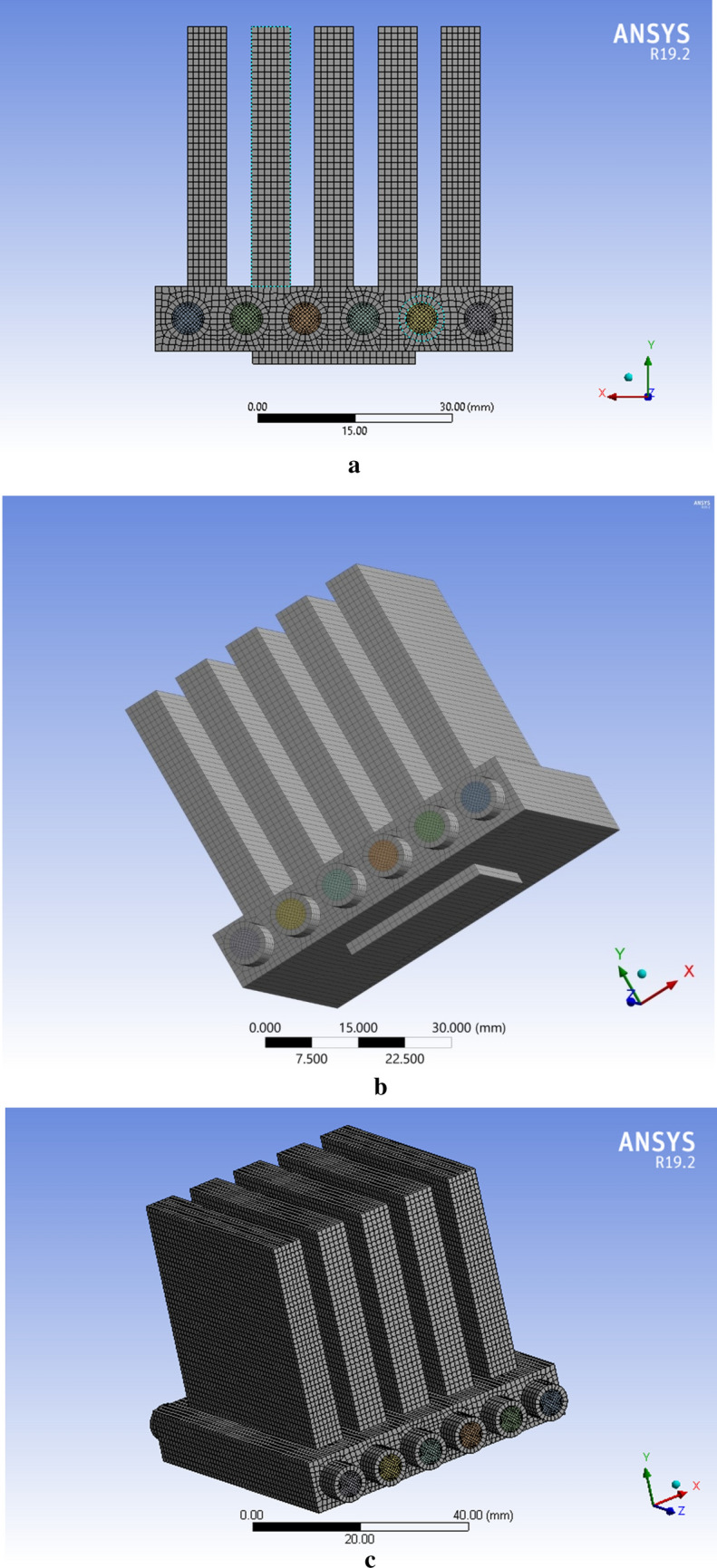
Computational mesh of the micropolar chip for cooling chip: (**a**) side view along the xy-plane, (**b**) bottom view, and (**c**) microchannel.

CFD modeling is now a well-established practice for computing complex issues in various modern engineering sectors because it is convenient. Here, the three-dimensional laminar flow of steady state from the laws of conservation of mass, momentum and energy is taken into account. A physical view of the problem under a heat sink with a microchannel is displayed in Figs. 2 and 3. According to the figure, six microchannels and five fins are installed with a chip to increase the cooling proficiency. The microchannel dimensions are width = 55 mm, length = 55 mm and height = 42 mm, as mentioned in Table 3. The microchannel in the heat sink and identical cross section of channels are shown in Fig. 4. To approximate the heat generation in the electronic chip, a uniform heat flux ($q^{\prime\prime} = 70\,\,{\text{W}}/{\text{cm}}^{2}$) is employed on the base wall surface of the channel heat sink. In this article, ${\text{TiO}}_{2}$ nanoparticles with a diameter of 35 mm were dispersed in water as a coolant. The computational package Fluent, which uses the finite volume method, is a popular program for computing fluid flows. Figure 5 are drawn for mash of microchannel heat sink in three different view (i) side view and five fins (ii) bottom view (iii) microchannel heat sink by utilizing ANSYS (i.e., CFD FLUENT) computational software.

**Table 3:** Properties of microchannel heat sink dimensions.

Dimension	Length (mm)	Width (mm)	Number of find (n)	Width of channel (mm)	Hydraulic diameter (mm)	Heat transfer area (mm^2^)
Size	55	50	5	6	6	0.0010362

The following governing equations are taken into account with consideration, such as laminar, incompressible and steady-state fluid flow, for the current issue:

Equation of Continuity

1 $$\partial_{x} u + \partial_{y} v + \partial_{z} w = 0,$$

Equations of Momentum are:

2 $$\rho_{f} \left( {u\partial_{x} u + v\partial_{y} u + w\partial_{z} u} \right) = - \partial_{x} p + \mu_{f} \left( {\partial_{xx} u + \partial_{yy} u + \partial_{zz} u} \right),$$

3 $$\rho_{f} \left( {u\partial_{x} v + v\partial_{y} v + w\partial_{z} v} \right) = - \partial_{y} p + \mu_{f} \left( {\partial_{xx} v + \partial_{yy} v + \partial_{zz} v} \right),$$

4 $$\rho_{f} \left( {u\partial_{x} w + v\partial_{y} w + w\partial_{z} w} \right) = - \partial_{z} p + \mu_{f} \left( {\partial_{xx} w + \partial_{yy} w + \partial_{zz} w} \right),$$

Heat equation for coolant:

5 $$\rho_{f} C_{pf} \left( {u\partial_{x} T_{f} + v\partial_{y} T_{f} + w\partial_{z} T_{f} } \right) = K_{f} \left( {\partial_{xx} T_{f} + \partial_{yy} T_{f} + \partial_{zz} T_{f} } \right),$$

Heat equation for solid phase:

6 $$k_{s} \left( {\partial_{xx} T_{s} + \partial_{yy} T_{s} + \partial_{zz} T_{s} } \right) = 0,$$

Here, $\rho_{f}$ indicates the density of the fluid, $\mu_{f}$ signifies the dynamic viscosity, $T_{f}$ designates the temperature of the coolant, $p$ denotes the coolant pressure, the specific heat capacity is indicated as $C_{pf}$ and $K_{f}$ is the thermal conductivity.

### Boundary conditions

The boundary layer flow equations were approached utilizing a commercial CFD package (Fluent 19.2). The volumetric concentration of ${\text{TiO}}_{2}$ nanoparticles dispersed in distilled water utilized as the input constraint was 1–3%, and the inlet temperature was $293.15\,{\text{K}}$ to the microchannel heat sink. The coolant used in this computation through the inlet is water and ${\text{TiO}}_{2}$/water nanoliquid, and its thermophysical characteristics are mentioned in Table 4. In this investigation, a single phase is considered. ANSYS-FLUENT (R19.2)^23^ is used for modeling the microchannel heat sink. Here, the nodes and elements of the microchannel heat sink are 174,216 and 421,945, respectively. A uniform heat flux $q^{\prime\prime} = 70\,\,{\text{W}}/{\text{cm}}^{2}$ is used on the base of the chip surface to simulate heat generation in electronic chips. No slip was implemented in any domain of the fluid.

**Table 4:** Thermophysical features of the base fluid (distilled water) and nanoparticles (${\text{TiO}}_{2}$).

Characteristics	Distilled water	${\text{TiO}}_{2}$ solid material
Density $\left( {{\text{kg}}\;{\text{m}}^{ - 3} } \right)$	997.1	4175
Specific heat $\left( {{\text{J}}\;{\text{kg}}^{ - 1} \;{\text{K}}^{ - 1} } \right)$	4179	692
Thermal conductivity $\left( {{\text{W}}/{\text{mK}}} \right)$	0.613	8.4
Dynamic Viscosity $\left( {{\text{N}}\;{\text{sm}}^{ - 2} } \right)$	0.001003	–
Mean Diameter $\left( {{\text{nm}}} \right)$	–	35

### Thermophysical characteristics of nanofluid

The thermophysical characteristics of the nanofluid, such as density ($\rho$), heat capacity ($C_{p}$), dynamic viscosity ($\mu$) and thermal conductivity ($k$), are addressed by^24^ (see Table 5).

**Table 5:** Thermophysical properties for nanofluid.

Characteristics	Symbols	Expressions for nanofluid
Density	$\rho$	$\rho_{nf} = \left( {1 - \phi } \right)\rho_{f} + \phi \rho_{p}$
Heat capacity	$C_{p}$	$C_{{p.nf}} = \left( {1 - \phi } \right)\left( {\rho C_{p} } \right)_{f} + \phi \left( {\rho C_{p} } \right)_{p} /\rho _{{nf}}$
Dynamic viscosity	$\mu$	$\mu_{nf} = \mu_{f} \left( {1 - \phi } \right)^{ - 2.5}$
Thermal conductivity	$k$	$$k_{nf} = \left( {{\begin{gathered} k_{p} + \left( {n - 1} \right)k_{f} \hfill \\ - \left( {n - 1} \right)\phi \left( {k_{f} - k_{p} } \right) \hfill \\ \end{gathered} \mathord{\left/ {\vphantom {\begin{gathered} k_{p} + \left( {n - 1} \right)k_{f} \hfill \\ - \left( {n - 1} \right)\phi \left( {k_{f} - k_{p} } \right) \hfill \\ \end{gathered} \begin{gathered} k_{p} + \left( {n - 1} \right)k_{f} \hfill \\ + \phi \left( {k_{f} - k_{p} } \right) \hfill \\ \end{gathered} }} \right. \kern-\nulldelimiterspace} \begin{gathered} k_{p} + \left( {n - 1} \right)k_{f} \hfill \\ + \phi \left( {k_{f} - k_{p} } \right) \hfill \\ \end{gathered} }} \right)k_{f}$$

### Data reduction

The heat transfer rate is obtained by:

7 $$Q_{nf} = m_{nf} C_{pnf} \left( {T_{out} - T_{in} } \right)_{nf} .$$

The coefficient of heat transfer is addressed as;

8 $$h_{nf} = Q_{nf} /A_{s} \left( {T_{s} - T_{nf} } \right).$$

The average Nusselt number is characterize as:

9 $$kNu_{av} = h_{av} .D_{h}$$

The Darcy friction factor along the microchannel heat sink is examined to compute the hydraulic loss of the microchannel flow^24^

10 $$f_{r} = \frac{{2D_{h} \Delta p}}{{\rho u^{2} L}}.$$

Here, $\Delta p$ indicates the difference in pressure between the inlet and outlet of the minichannel heat sink $f_{r}$ is the friction factor and $L$ is the length of the microchannel.

Based on hydraulic diameter, the Reynolds number is computed as^25^,

11 $$\mu Re = \rho u \cdot D_{h}$$

The overall thermal resistance is determined by

12 $$R_{th} = \frac{{T_{\max } - T_{in} }}{{Q_{in} }}$$

The Arrhenius expression is addressed by^26^

13 $$AF = e^{{\left( {\frac{Ea}{k}\left( {\frac{T}{{T_{use} }} - \frac{1}{{T_{stress} }}} \right)} \right)}}$$

The Failure rate^26^ is obtained by following

14 $$FIT = \lambda_{FIT} = \lambda_{hours} \times 10^{9}$$

The MTTF (i.e., Mean Time to Failure) is calculated as^26^:

15 $$FTTF_{hours} = 1/\lambda_{FIT} = \lambda_{hours} .$$

## Numerical analysis/CFD methodology

In this investigation, commercial ANSYS-FLUENT (R19.2) CFD software was utilized to compute numerically governing equations. The mesh of the microchannel for the electronic ship is computed at fine quality by utilizing ANSYS-FLUENT (R19.2) (see Fig. 5). Here, the microchannel heat sink for an electronic chip utilizing a ${\text{TiO}}_{2}$/distilled water nanofluid is illustrated by a single phase with uniform heat flux $q^{\prime\prime} = 70\,\,{\text{W}}/{\text{cm}}^{2}$ affected on the bottom chip surface. The governing flow equations are integrated utilizing FVM with CFD ANSYS FLUENT software. The computations were based on the pressure correction technique utilizing the SIMPLE scheme.

## Results and discussion

In this numerical study, the heat transfer in electronic chip in six circular channel heat sink can be divided into two based on the mechanisms namely, simple water and ${\text{TiO}}_{2}$/water nanofluid. The thermal transfer rate, Nusselt number, Darcy friction factor, Reynolds number, thermal resistance, and wall temperature are discussed and compared with the outcomes of water. Nanopowder of ${\text{TiO}}_{2}$ is that this particular nanofluid is unique as compared with other nano fluid especially when it comes to heat transfer. The computations address dissimilar values of the volume friction of ${\text{TiO}}_{2}$/water nanoliquids, such as $\phi = 1\%$, $\phi = 2\%$ and $\phi = 3\%$. The single-phase ${\text{TiO}}_{2}$/water nanofluid in the microchannel heat sink is studied numerically with uniform heat flux $q^{\prime\prime} = 70\,\,{\text{W}}/{\text{cm}}^{2}$ on the base of the chip.

### Velocity profile

**Figure 6:**
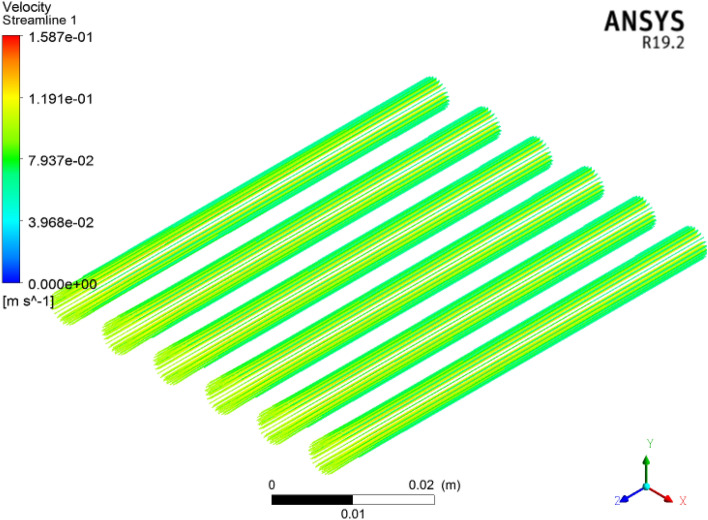
Velocity in micro channel heat sink.

**Figure 7:**
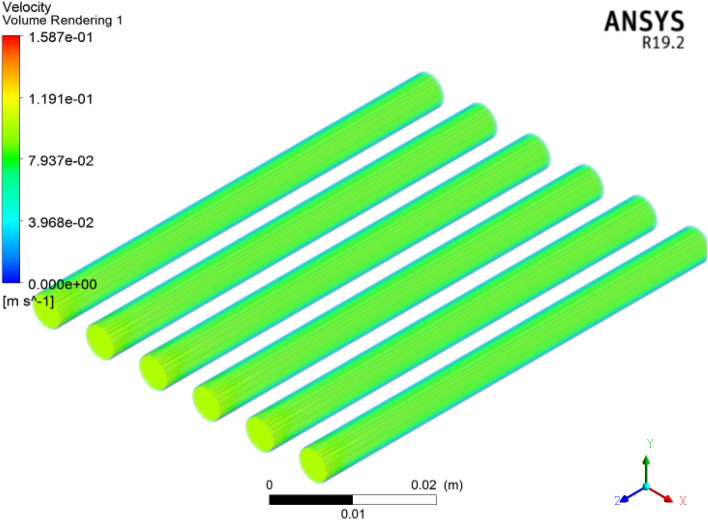
Cross-sectional view of velocity in microchannels by ANSYS.

The velocity field across the microchannel heat sink for the ${\text{TiO}}_{2}$/water nanofluid is plotted in Fig. 6. The variation in the velocity field $1587\,\,{\text{ms}}^{ - 1}$ is obtained at the $3\%$ volume friction of the nanofluid. It can be observed that the thermophysical properties are altered with respect to volume friction. Here, we found that velocity decreases as the volume friction in the nanofluid increases. The cross-sectional view of velocity across inlet to outlet is displayed in Fig. 7. The velocity decreases at the inlet to outlet faces for each volume friction under the Reynolds number. Furthermore, the density and viscosity of the nanofluid increases via superior volume friction impact under the same Reynolds number.

### Heat transfer

**Figure 8:**
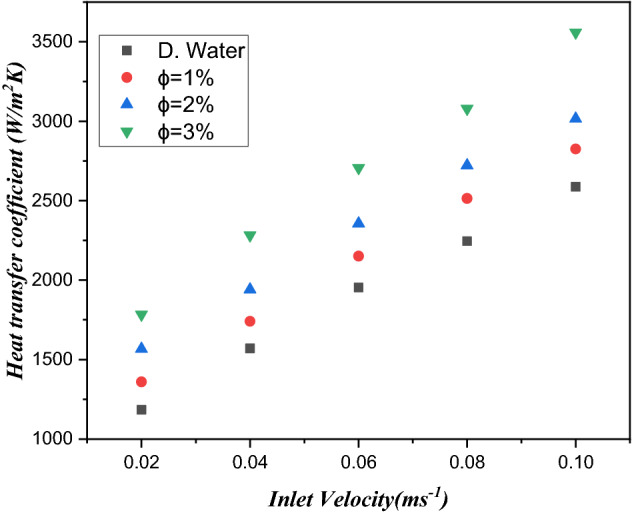
Estimation of the heat transfer coefficient against inlet velocity with ${\text{TiO}}_{2}$/water nanofluid and distilled water in the circular channel.

**Figure 9:**
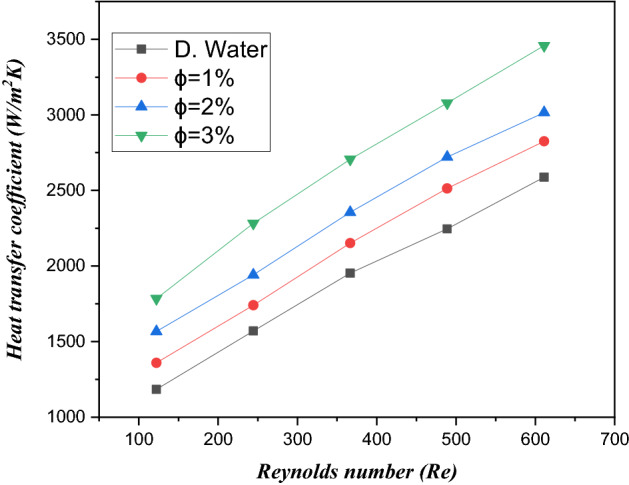
Estimation of the heat transfer coefficient against the Reynolds number in a minichannel heat sink for a microelectronic chip with ${\text{TiO}}_{2}$/water nanofluids and distilled water.

The major aim of the cooling system is to decrease the surface temperature of the heat sink in minichannels with electronic chips. Figure 8 signifies the variation of heat transfer coefficient as a function of inlet velocity with distinguished amount of volume friction of ${\text{TiO}}_{2}$/water nanofluid such as $\phi = 1\%$, $\phi = 2\%$ and $\phi = 3\%$. The heat transfer coefficient has a direct direction with the inlet velocity of the fluid. Therefore, the heat transfer coefficient improves with increasing inlet velocity of the fluid. The coefficient of heat transfer is observed from $12.09$ to $14.02\%$ at a volume friction of ${\text{TiO}}_{2}$/water nanofluid from 1 to $3\%$. Figure 9 illustrates the heat transfer coefficient against the Reynolds number for distilled water and three different amounts of volume friction $1\%$ to $3\%$. A larger Reynolds number has a larger heat transfer coefficient, with the volume friction distinguishing values from 1 to $3\%$ of the ${\text{TiO}}_{2}$/water nanofluid in the microchannel heat sink. Furthermore, the heat transfer rate is larger from $12.09$ to $14.02\%$ in the case of ${\text{TiO}}_{2}$/water nanofluids compared to water. This improvement due to the superior merging of ${\text{TiO}}_{2}$ particles and collision of particles as a result of heat efficiency carries out more heat proficiency.

**Figure 10:**
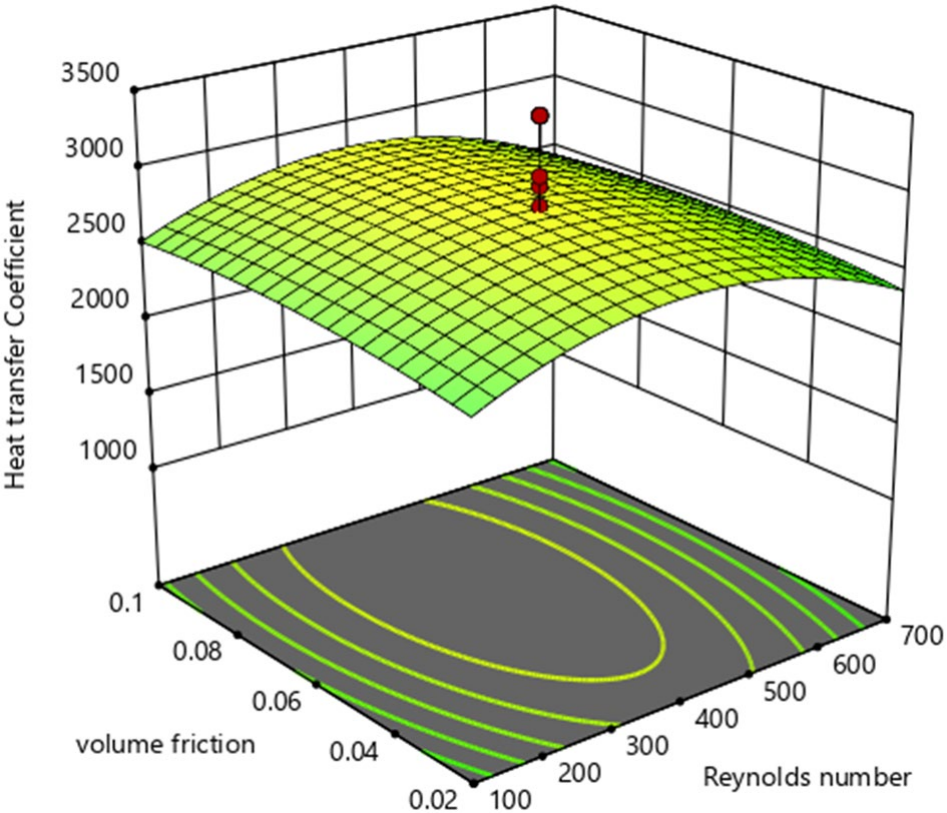
3D graphs of heat transfer coefficient vs. volume friction and Reynolds number.

**Figure 11:**
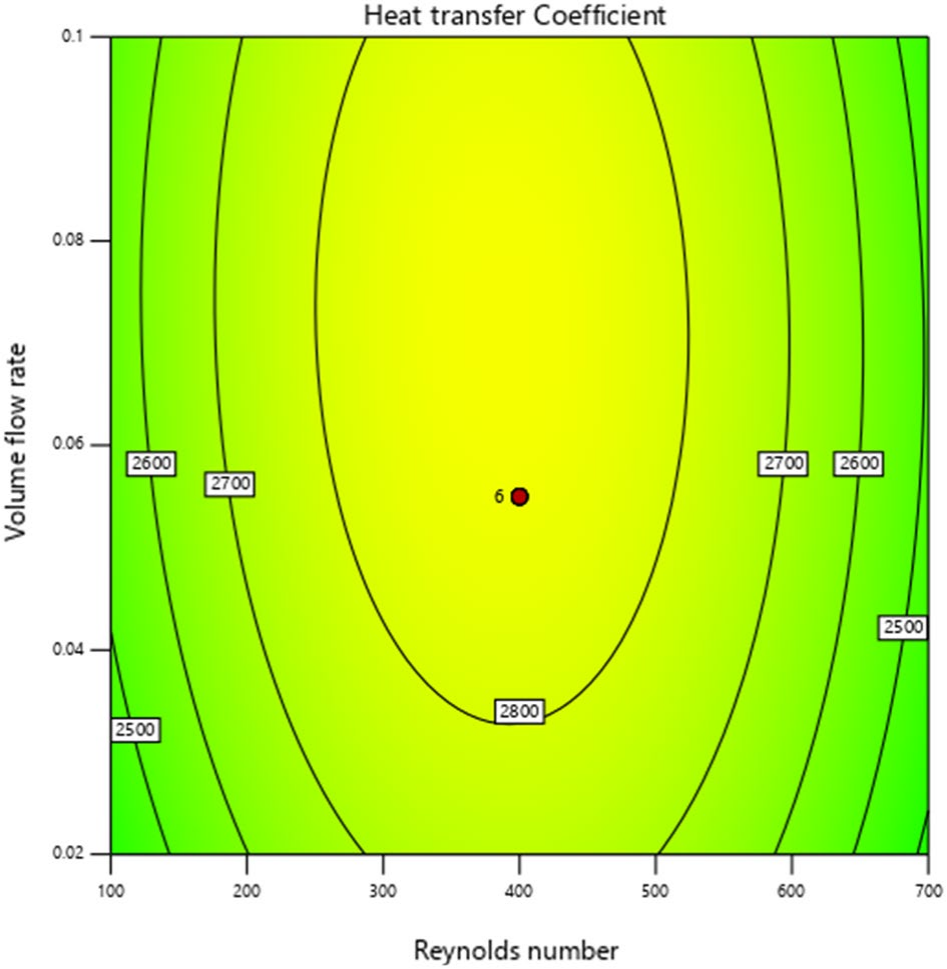
Heat transfer coefficient contour diagram influenced by volume friction and Reynolds number.

The 3D graph impacts of HTC w.r.t active parameters are plotted in Fig. 10. Similarly, Fig. 11 demonstrates the contour diagram of the heat transfer coefficient (HTC) to indicate the largest and smallest amount.

### Nusselt number

**Figure 12:**
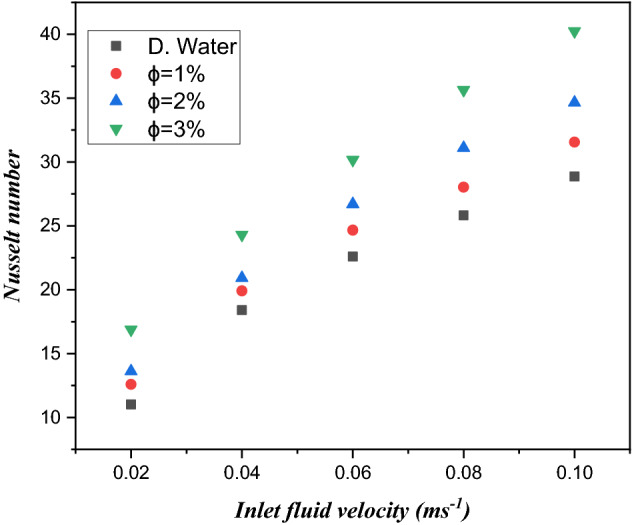
Estimation of the Nusselt number against inlet fluid velocity across the microchannel for an electronic chip heat sink with ${\text{TiO}}_{2}$/water nanofluid and distilled water.

The significance of inlet fluid velocity on the Nusselt number with three different values of ${\text{TiO}}_{2}$/water nanofluid volume friction $\phi = 1\%$, $\phi = 2\%$ and $\phi = 3\%$ is displayed in Fig. 12. We observed that the use of ${\text{TiO}}_{2}$/water nanoliquids in microchannel heat sinks with heat flux escalates the thermal transfer rate. As mentioned earlier, the use of different volume friction ($\phi = 1\%$, $\phi = 2\%$ and $\phi = 3\%$) of nanofluids leads to an enhanced Nusselt number. From the figure, it can be noticed that the Nusselt number is an improving function of inlet fluid velocity.

**Figure 13:**
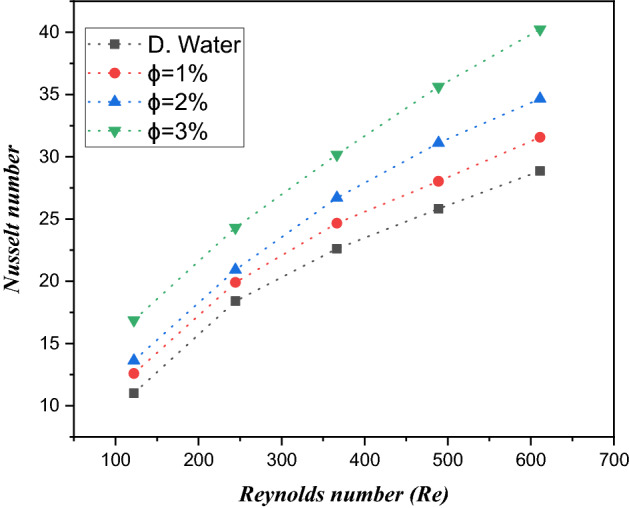
Estimation of the Nusselt number against the Reynolds number in a minichannel heat sink with ${\text{TiO}}_{2}$/water nanofluid and distilled water.

Figure 13 is captured to determine the trend of the Reynolds number against the Nusselt number with volume friction ($\phi = 1\%$, $\phi = 2\%$ and $\phi = 3\%$) of ${\text{TiO}}_{2}$/water nanofluids in a microelectronic chip with six microchannel heat sinks. From the CFD analysis, we found that the Nusselt number is improved by escalating the values of the Reynolds number. Furthermore, the Nusselt number is, $12.17\%$, $12.39\%$ and $14.01\%$ larger than water for different $\phi = 1\%$, $\phi = 2\%$ and $\phi = 3\%$ ${\text{TiO}}_{2}$/water nanoliquids, respectively. Physically, the convective thermal transfer current is improved due to the presence of a better Reynolds number. Therefore, the Nusselt number is more effective for ${\text{TiO}}_{2}$/water nanoliquids than water.

**Figure 14:**
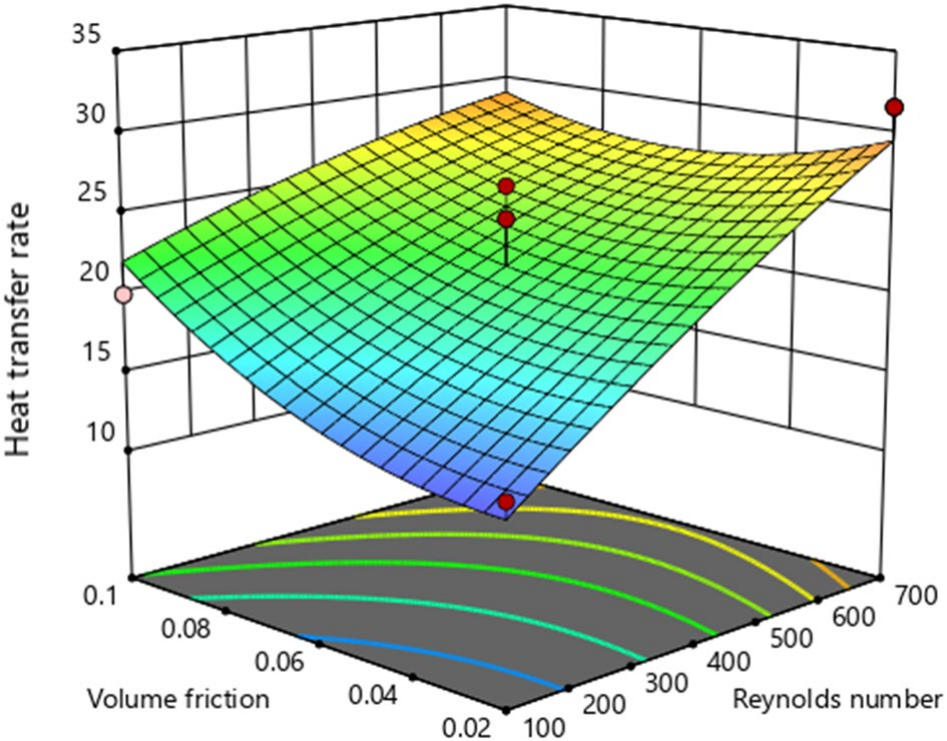
3D graphs of heat transfer rate (Nusselt number) versus volume friction and Reynolds number.

**Figure 15:**
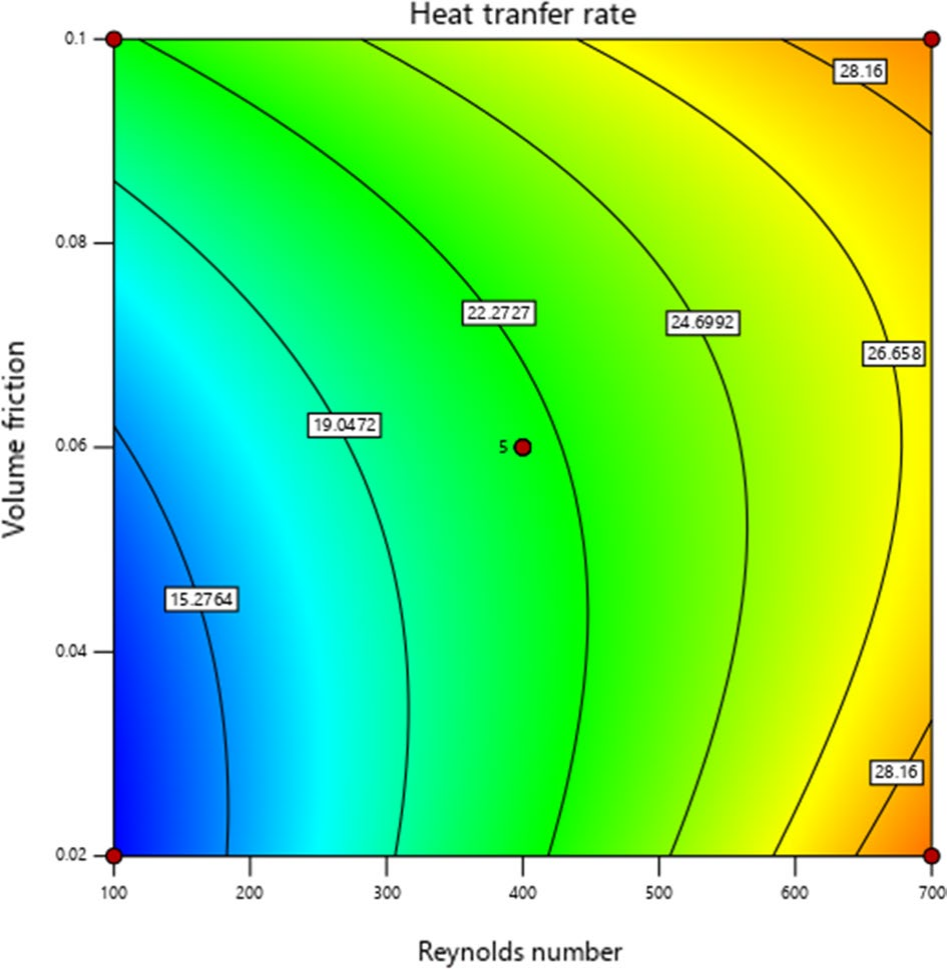
Heat transfer rate (Nusselt number) contour diagram influenced by volume friction and Reynolds number.

Figure 14 illustrates a 3D plot of the heat transfer rate (HTR) against the volume friction and Reynolds number. Here, we noticed that HTR is enhanced when the Reynolds number is boosted for each volume friction. Similarly, the contour of HTR versus active parameters is captured in Fig. 15.

### Thermal resistance

**Figure 16:**
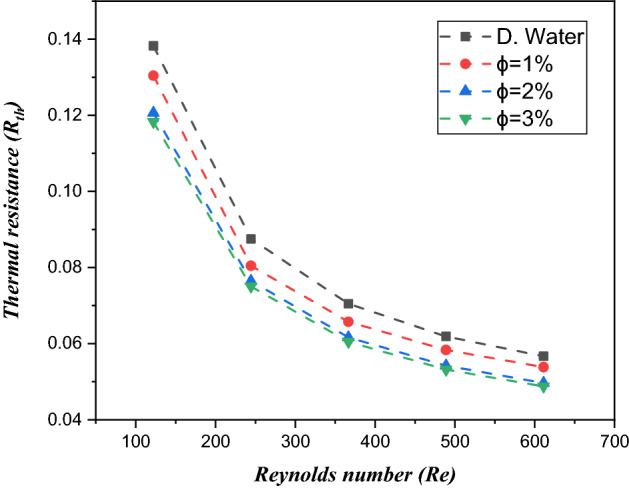
Estimation of the thermal resistance against the Reynolds number in a minichannel heat sink with ${\text{TiO}}_{2}$/water nanofluid and distilled water.

Figure 16 demonstrates the thermal resistance values for dissimilar values of ${\text{TiO}}_{2}$/water nanofluid volume fractions versus Reynolds number. Larger values of Reynolds cause a reduction in thermal resistance due to volume friction ($\phi = 1\%$, $\phi = 2\%$ and $\phi = 3\%$) of the nanofluid. It can be observed that $\phi = 3\%$ ${\text{TiO}}_{2}$/water nanofluid volume friction demonstrated $16.25\%$ less thermal resistance than water. Physically, the reduction thermal resistance is due to the significantly greater velocity when the Reynolds number is larger. The larger velocity of fluid depresses the thermal resistance among the fluid particles, and larger nanofluids have more heat proficiency. This leads to increased thermal transport, which is inversely proportional to convective thermal resistance.

### Friction factor

**Figure 17:**
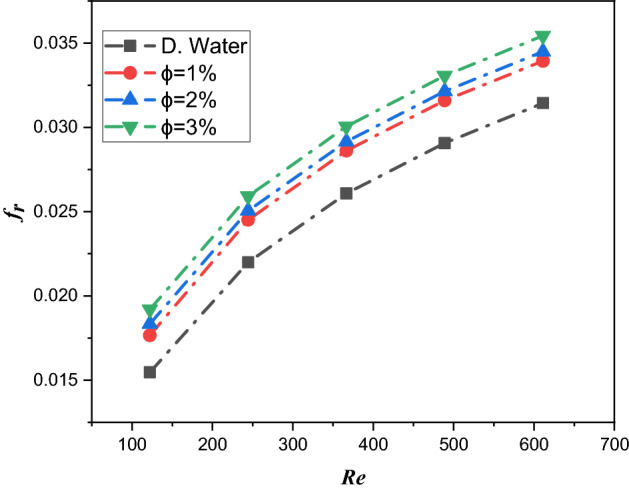
Estimation of the friction factor against the Reynolds number in a microchannel heat sink with ${\text{TiO}}_{2}$/water nanofluids and distilled water.

Figure 17 elucidates the friction factor of nanofluid aspects for ${\text{TiO}}_{2}$/water nanofluid volume friction ($\phi = 1\%$, $\phi = 2\%$ and $\phi = 3\%$) via the Reynolds number. The friction factor of the nanoliquid in the microelectronic six-channel heat sink is reduced by enhancing the Reynolds number. Physically, due to the larger Reynolds number, the velocity is boosted, and the velocity has an opposite relation with the friction factor; therefore, the friction factor of the nanofluid declines with a greater Reynolds number.

### Wall temperature

**Figure 18:**
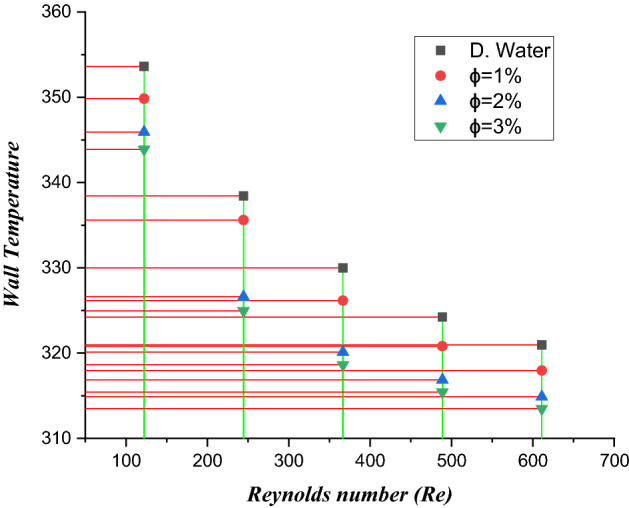
Estimation of the Wall temperature against the Reynolds number in a microchannel heat sink with ${\text{TiO}}_{2}$/water nanofluid and distilled water.

**Figure 19:**
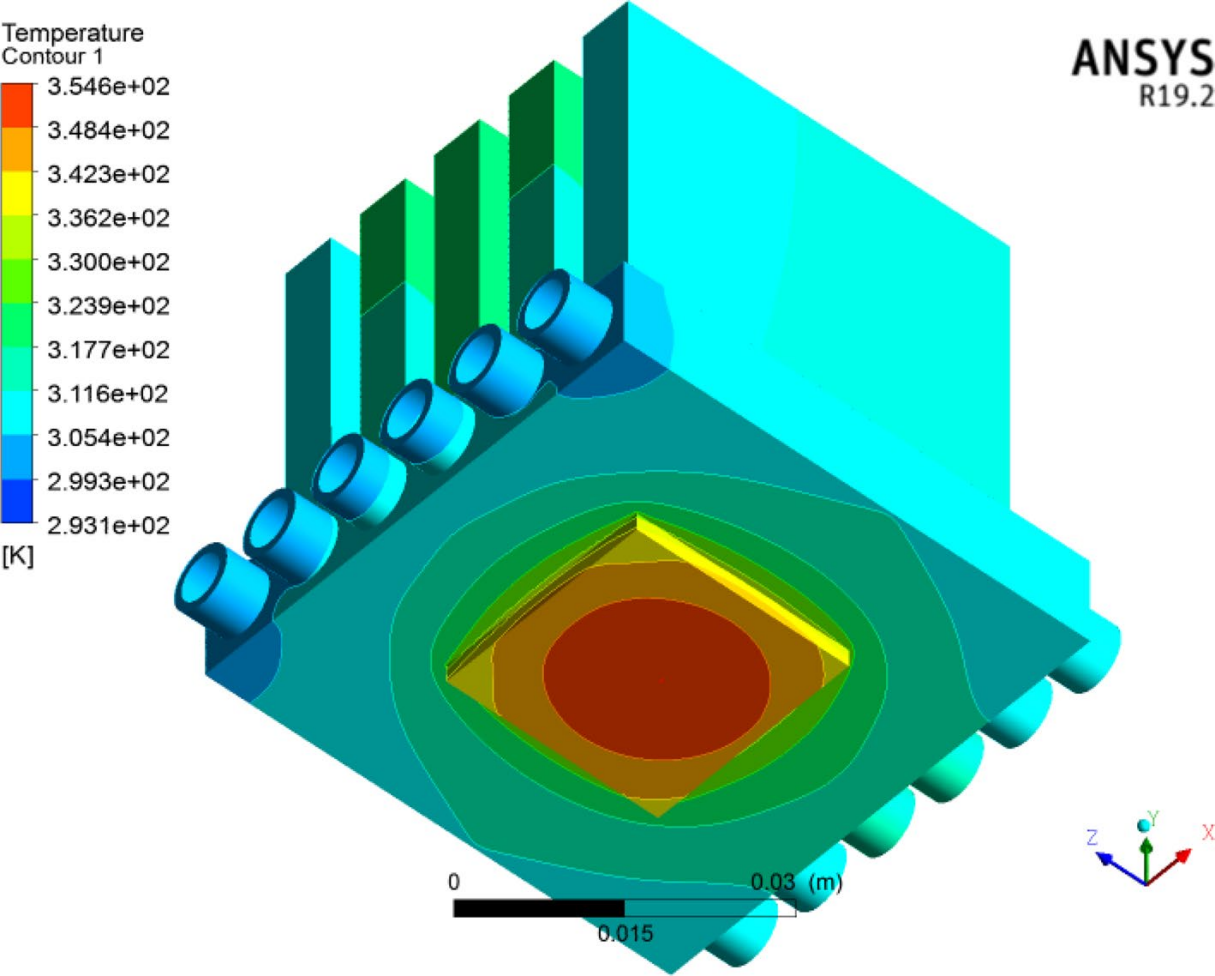
Temperature contour for a microelectronic chip in a microchannel heat sink with a nanofluid utilizing ANSYS.

The wall temperature estimations at the bottom side of the heat sink for dissimilar volume friction ($\phi = 1\%$, $\phi = 2\%$ and $\phi = 3\%$) of ${\text{TiO}}_{2}$/water nanofluids in a microelectronic chip with six mini channels via the Reynolds number are shown in Fig. 18. Here, it can be noticed that the wall temperature diminishes via a greater Reynolds number for different volume friction ($\phi = 1\%$, $\phi = 2\%$ and $\phi = 3\%$). Figure 19 shows the thermal/temperature contour in the minichannel heat sink for the nanofluid. From the figure, we observed that the wall temperature of the six circular microchannels declines significantly when utilizing coolant. The wall temperature found was $3546\,\,{\text{K}}$. The largest heat transfer rates are examined for concentration volume friction from 1 to $3\%$. The outcome depicts that improving the heat transfer coefficient enhances the thermophysical characteristics of the dispersed nanomaterials compared to the host fluid. Thus, ${\text{TiO}}_{2}$ a larger heat efficiency of/water nanoliquids improves heat transfer along the microchannel heat sink.

### Pressure drop

**Figure 20:**
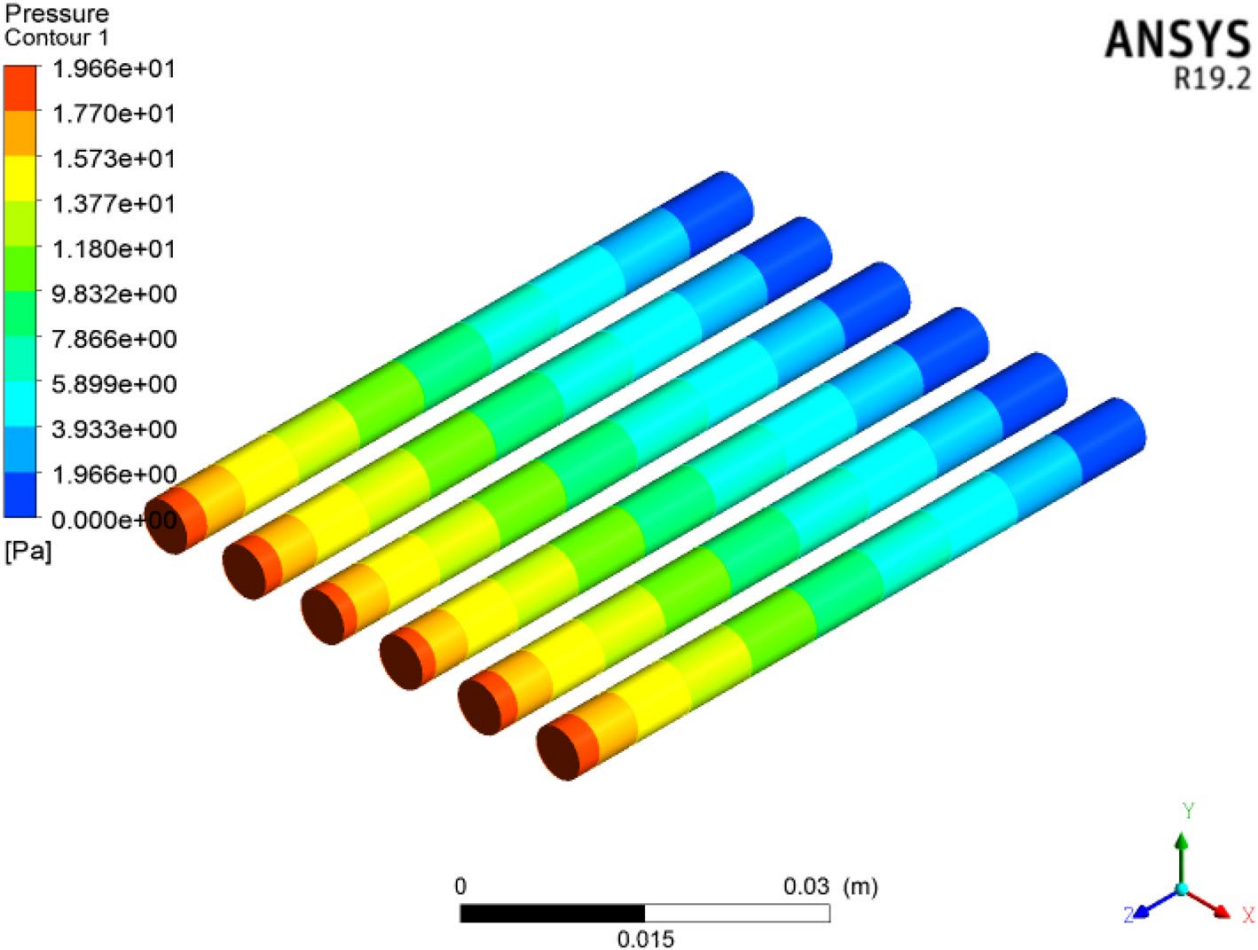
Contour of pressure in six circular microchannels.

**Figure 21:**
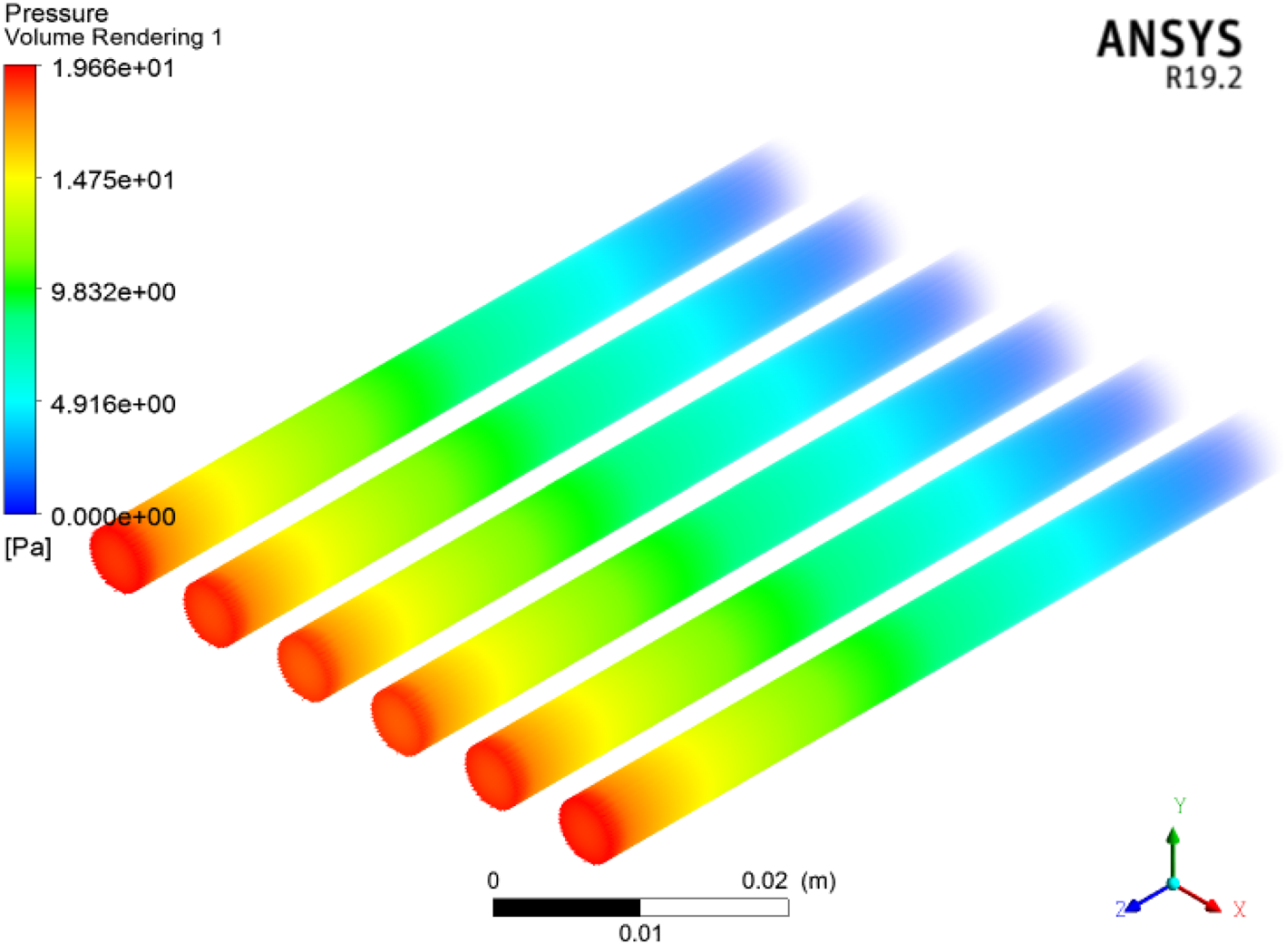
Cross-sectional observation of pressure in six circular microchannels.

The pressure drop across the inlet to outlet face of the six circular microchannel heat sinks from adopting ANSYS-FLUENT is shown in Fig. 20. From the CFD analysis, it can be noticed that the pressure drop is enhanced for each concentration of coolant. Here we acquire $1996\,\,{\text{Pa}}$ pressure. Figure 21 signifies the pressure drop contour across the inlet to outlet of the six microchannel heat sinks displayed in the cross section utilizing ANSYS-(R19.2) FLUENT. In these figures, we clearly observed two regions with smaller temperature and pressure (blue color) in the inlet of the circular channel heat sink and in the outlet face of the channel greater temperature and pressure drop (red color). Due to the frictional consequences of the nanoparticles and the surface of the channel, the pressure drop increases. Therefore, the pressure drop of the ${\text{TiO}}_{2}$/water nanoliquid improved with an increase in volumetric concentration friction significance. Here we obtained $1996\,\,{\text{Pa}}$ pressure.

### Wall heat flux

**Figure 22:**
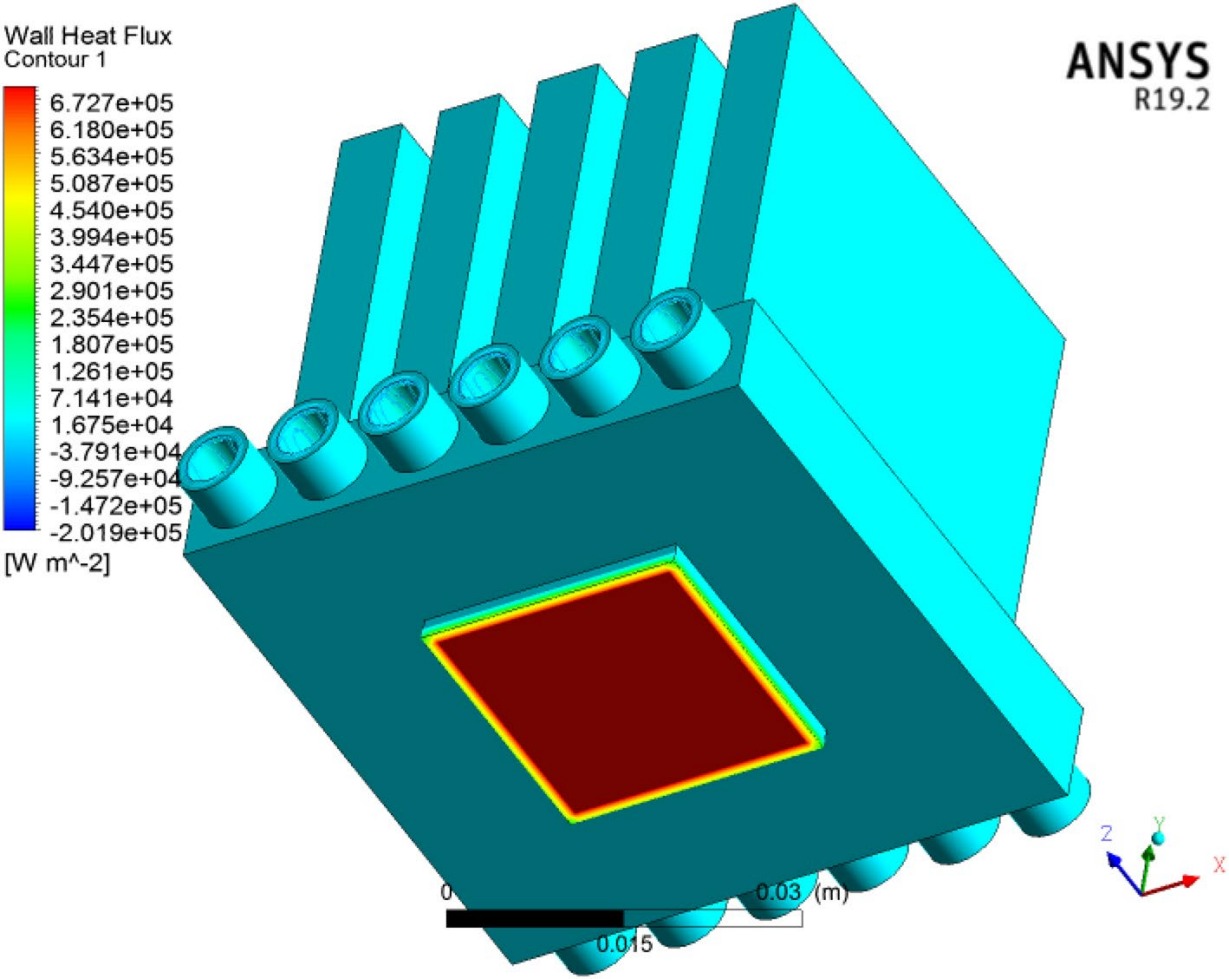
Wall heat flux significance for microelectronic chips in microchannel heat sinks with nanofluids by using ANSYS.

**Figure 23:**
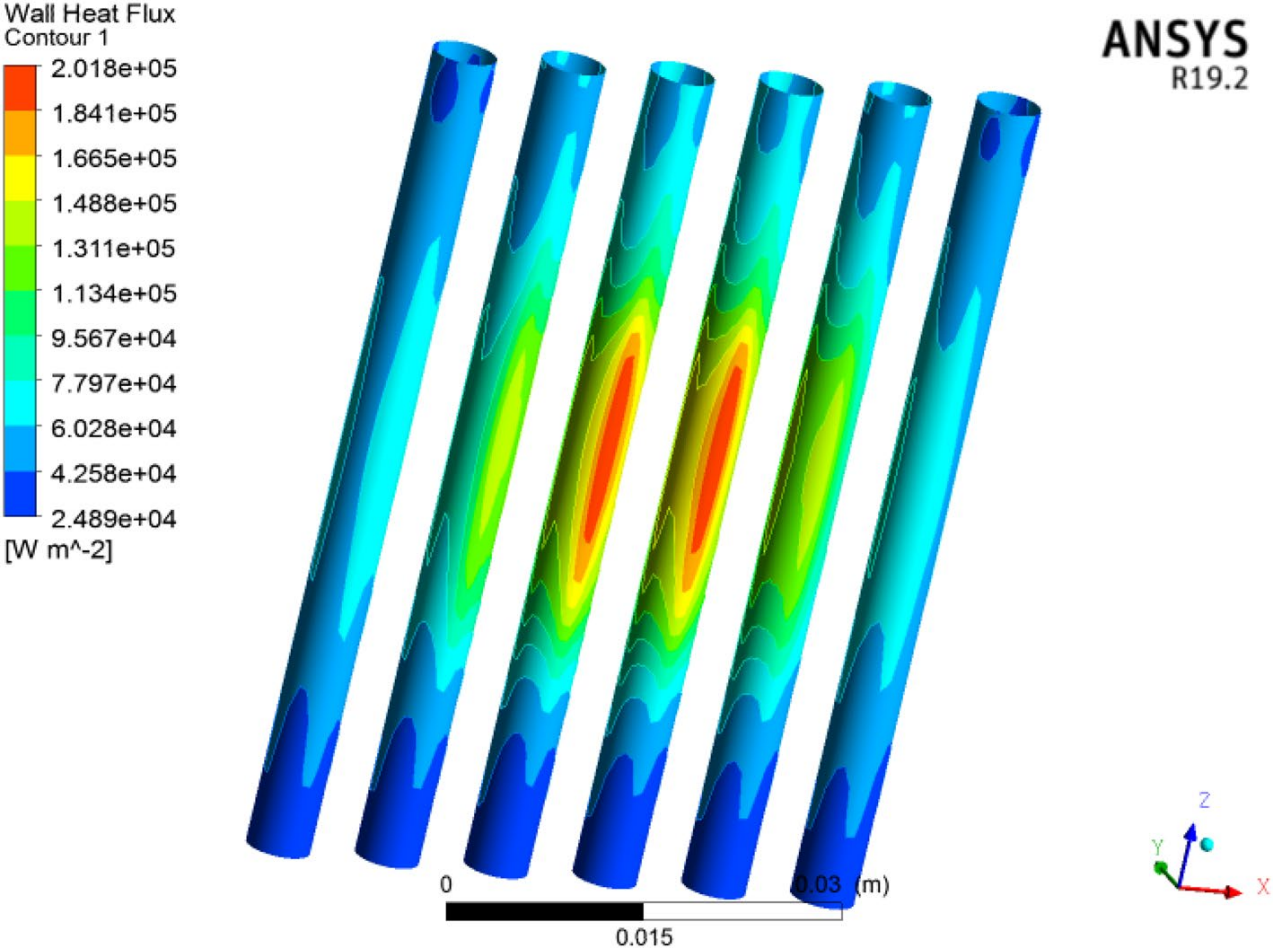
Wall heat flux contour in six circular microchannels.

**Figure 24:**
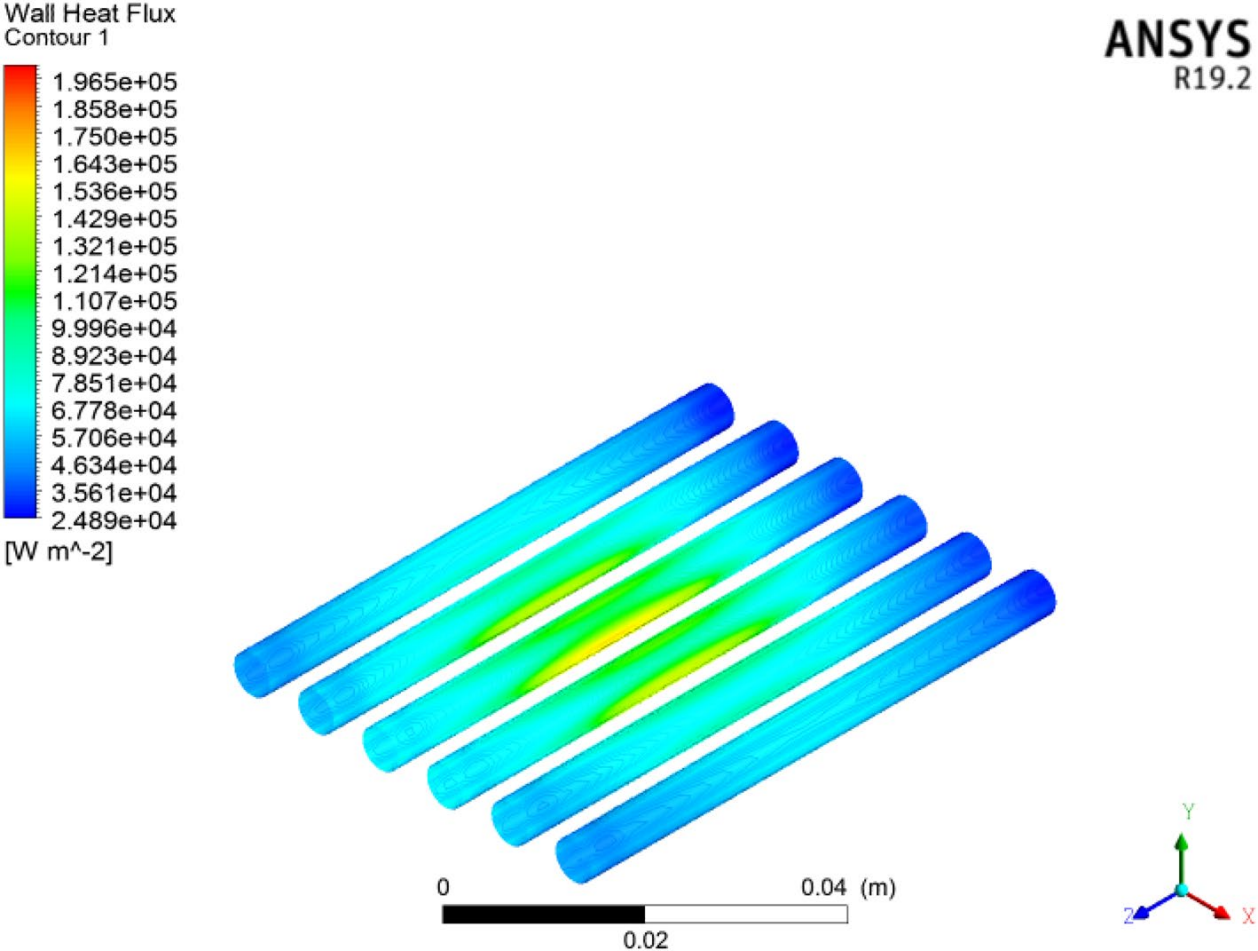
Cross-sectional Schmidt of the wall heat flux contour in the microchannel heat sink**.**

Figure 22 shows the wall heat flux in the microchannel heat sink for each volumetric concentration. The heat flux acts at the underside surface of the microchannel heat sink to compute the heat generation in the microelectronic chip. Here we acquired $6727\,\,{\text{Wm}}^{ - 2}$ heat flux. Figure 23 clearly shows that the heat flux is clearly applied on the bottom of the microchannels in the electronic chip. We observed that heat flux is increased at each concentration of cooling. The wall heat flux acquired was $2018\,\,\,{\text{Wm}}^{ - 2}$. The heat flux across the inlet to outlet faces of six circular microchannels; a cross-sectional view is shown in Fig. 24. The heat flux is improved for larger concentrations of coolant. Heat flux has a direct relation with thermal conductivity. Larger heat efficiency was carried out to improve the heat transfer. The ${\text{TiO}}_{2}$/water nanofluid has high heat transfer across microchannels.

## Conclusions

In this study, CFD analysis was conducted to scrutinize the numerical cooling performance of circular minichannel heat sinks with different volume friction ($\phi = 1\%$, $\phi = 2\%$ and $\phi = 3\%$) using ${\text{TiO}}_{2}$/water nanofluids and water for electronic chips. The computational ANSYS-FLUENT (R19.2) Package is used to observe the CFD analysis. The flow governing equations are approached numerically by (FVM) with the ANSYS-FLUENT package. Based on the outcomes, it was observed that a noteworthy increment in the heat transfer coefficient was obtained by using nanofluids as coolants w.r.t water. It is scrutinized that the heat transfer coefficient is escalated by enlarging the inlet velocity. The HTC is improved $12.09\%$ to $14.02\%$ with concentration $\phi = 1\%$, $\phi = 2\%$ and $\phi = 3\%$ of nanofluid compared to water. The Nusselt number is amplified via larger estimations of the Reynolds number and volume friction. It is noted that the thermal resistance and friction factor decrease when the nanoparticle concentration increases under the Reynolds number. These effects occur due to the larger thermal efficiency of the nanofluid and random motion of nanoparticles. Nanofluids used in microchannel heat sinks enhance the efficiency of electronic chip cooling compared to distilled water.
